# Automatic analysis of nuclear features reveals a non-tumoral predictor of tumor grade in bladder cancer

**DOI:** 10.1186/s13000-024-01501-5

**Published:** 2024-06-08

**Authors:** Ibrahim Fahoum, Shlomo Tsuriel, Daniel Rattner, Ariel Greenberg, Asia Zubkov, Rabab Naamneh, Orli Greenberg, Valentina Zemser-Werner, Gilad Gitstein, Rami Hagege, Dov Hershkovitz

**Affiliations:** 1 Institute of Pathology, Tel-Aviv Sourasky Medical Center, Tel-Aviv, Israel; 2 Institute of Pathology, Rabin Medical Center, Petah-Tikva, Israel; 3 Faculty of Medicine, Tel-Aviv University, Tel-Aviv, Israel

## Abstract

**Background & objectives:**

Tumor grade determines prognosis in urothelial carcinoma. The classification of low and high grade is based on nuclear morphological features that include nuclear size, hyperchromasia and pleomorphism. These features are subjectively assessed by the pathologists and are not numerically measured, which leads to high rates of interobserver variability. The purpose of this study is to assess the value of a computer-based image analysis tool for identifying predictors of tumor grade in bladder cancer.

**Methods:**

Four hundred images of urothelial tumors were graded by five pathologists and two expert genitourinary pathologists using a scale of 1 (lowest grade) to 5 (highest grade). A computer algorithm was used to automatically segment the nuclei and to provide morphometric parameters for each nucleus, which were used to establish the grading algorithm. Grading algorithm was compared to pathologists’ agreement.

**Results:**

Comparison of the grading scores of the five pathologists with the expert genitourinary pathologists score showed agreement rates between 88.5% and 97.5%.The agreement rate between the two expert genitourinary pathologists was 99.5%. The quantified algorithm based conventional parameters that determine the grade (nuclear size, pleomorphism and hyperchromasia) showed > 85% agreement with the expert genitourinary pathologists. Surprisingly, the parameter that was most associated with tumor grade was the 10th percentile of the nuclear area, and high grade was associated with lower 10th percentile nuclei, caused by the presence of more inflammatory cells in the high-grade tumors.

**Conclusion:**

Quantitative nuclear features could be applied to determine urothelial carcinoma grade and explore new biologically explainable parameters with better correlation to grade than those currently used.

## Introduction

Urothelial carcinoma of the urinary bladder is the fourth most common cancer in men and the twelfth in women [1]. The histologic grade is one of the most important prognostic and predictive factors in urothelial carcinoma [2, 3].The 2004/2016 WHO classification separates urothelial carcinomas into two groups; low grade and high grade. In 2022, the consensus meeting of the International Society of Urological Pathology (ISUP) has issued new recommendations to refine grading of urothelial carcinoma into a 3-tier scheme with the division of WHO 2004/2016 high grade into clinically relevant categories (grade 2 and grade 3) [4, 5], and to report the presence of mixed low and high grade tumors [6].High grade urothelial carcinoma (HGUC) is associated with higher risks of disease recurrence and progression compared to low grade urothelial carcinoma (LGUC) [7, 8]. The grading process is based on the evaluation of several cytological and architectural parameters that include the nuclear size, hyperchromasia, pleomorphism and loss of polarity [9]. LGUC are characterized by overall mild nuclear enlargement, scattered cells with hyperchromatic nuclei, mild loss of nuclear polarity and mild variation in nuclear size. On the other hand, the clinically more aggressive HGUC display more significant nuclear size variation, nuclear hyperchromasia and loss of polarity. Differentiating between low and high grade tumors has a significant impact on treatment and prognosis. Nevertheless, it can be a challenging task for pathologists. Urothelial carcinomas exist along a morphologic spectrum and may therefore show intratumoral grade heterogeneity [10, 11]. Another problem is the interobserver variability among pathologists [12]. The nuclear features upon which grading is based are not numerically measured. These histologic characteristics are subjectively evaluated, hence they may be interpreted differently by pathologists.

Digital pathology and image analysis has the potential to increase the quality of urothelial carcinoma grading. Artificial intelligence (AI) based algorithms have been applied in several medical fields such as radiology [13] and pathology [14–16]. Promising results have already been seen in AI assisted Gleason grading for prostate cancer [17]. The purpose of this study is to assess the value of a computer-based image analysis tool for identifying predictors of urothelial carcinoma grade.

## Methods

### Patients and samples

Hematoxylin and Eosin stained slides were scanned with the Philips UFS scanner (Koninklikje Philips, Amsterdam, The Netherlands). High magnification images (40x) were captured from 20 TURBT cases: 10 cases of LGUC and 10 cases of non-invasive HGUC that were collected between September 2021 and December 2021. A Total of 200 images (100 LGUC, 100 HGUC) were captured. This study was approved by the institutional ethics committee at Tel-Aviv Sourasky Medical Center.

### Grading by pathologists

**Fig. 1:**
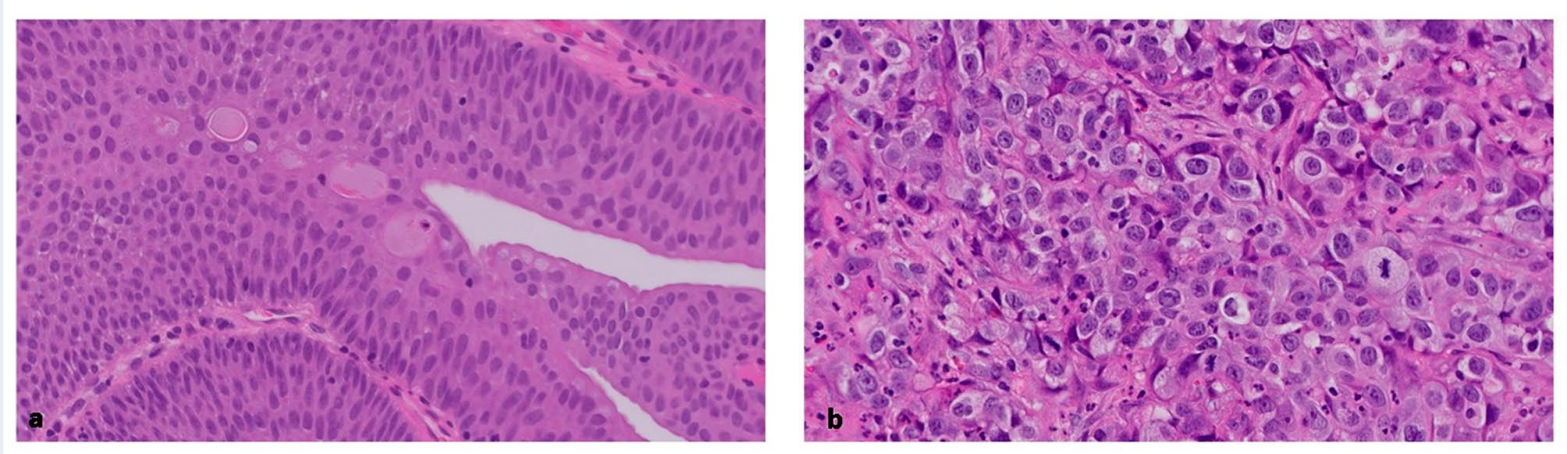
Examples of tumors with score 1 (**a**), the lowest grading score, and score 5 (**b**), the highest grading score

Five pathologists graded each tumor image. To increase the resolution, the grading was on a scale of grade 1, the lowest grade, to grade 5, the highest grade (Fig. 1). In addition, two expert genitourinary pathologists (I.F. and G.G.) graded the tumors in a similar manner. Both expert pathologists are dedicated genitourinary pathologists with over 5 years of experience in genitourinary pathology. The grading by pathologists was performed by assessing all the conventional histologic features that determine the tumor grade, and these include nuclear pleomorphism, hyperchromasia, loss of polarity and crowding. All images were anonymous and the pathologists had no access to personal information of participants.

### Development of automatic nuclear segmentation tool

We developed an algorithm that accurately segments all the nuclei in the H&E images independent of the type of tissue. Tumor areas were segmented in all images using the nucleus segmentation tool. We collected different properties on over 140,000 nuclei in the set of 200 images. The properties collected included geometrical parameters such as area, circumference, diameter, color intensity characteristics such as average red, blue, green, texture information such as granularities and neighborhood analysis such as distance to closest nuclei. In total we collected 40 properties for each nucleus, so the total collected data is a table of approximately 140,000 × 40 ~ 5 Million numbers describing 200 different images graded by five pathologists.

We used these numbers in order to develop an algorithm that, given an image, extract 40 properties for each nucleus in the image and based on that automatically grade the tumor.

### Analysis of nuclear parameters

Based on the grading scores of the pathologists, the images were divided into three groups: ' group 1’ that includes all the images that were given a grade score ‘1’ by at least 4 pathologists; ‘group 5’ has all the images with grade score ‘5’ by 4 pathologists or more; and ‘group 3’ includes the images with an average grade score between 2.5 and 3.5. A t-test was preformed to compare between the three groups in each one of the nuclear parameters, and those with *p* < 0.01 were selected. Based on the average of each parameter in groups 1 and 5, a two-points-based linear function was established to predict the expected grade score of the pathologists. The computer-based score for each nuclear parameter was given based on the appropriate linear function.

### Validation cohort

The algorithm was further validated on additional 200 images (100 LGUC, 100 HGUC) from new 20 TURBT cases: 10 cases of LGUC and 10 cases of non-invasive HGUC.

## Results

**Fig. 2:**
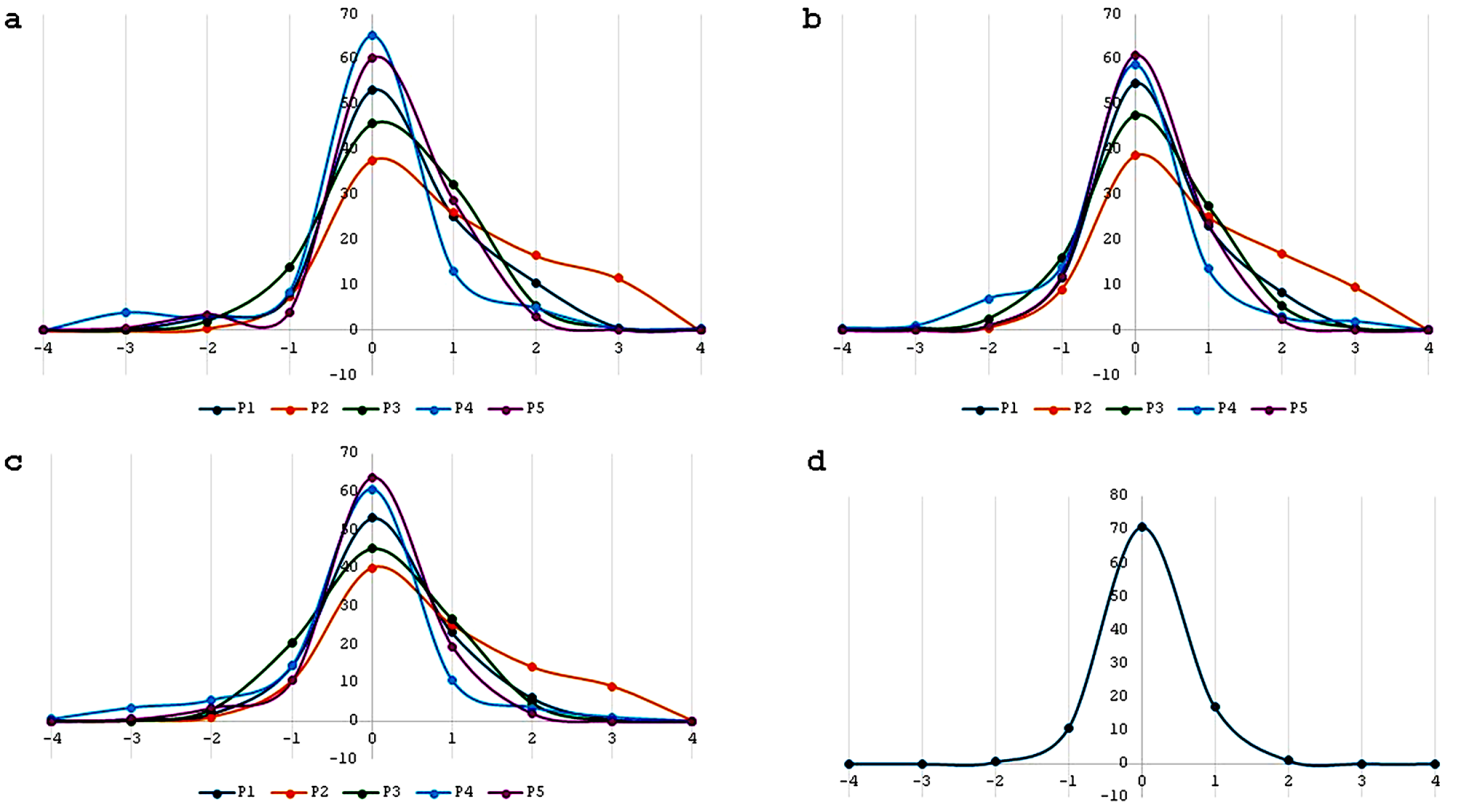
**a**-**c**: The distribution of the grading scores of the five pathologists in comparison with the first (**a**), second (**b**) expert genitourinary pathologists, and the mean of the two experts (**c**). The horizontal scale represents the delta between the score of the pathologists and the expert pathologist. The agreement rates ranges between 88.5–97.5%. The graphs of the 5 pathologists “shifted to the right”, a finding that indicates that the pathologists tended to overgrade tumors in comparison to the expert genitourinary pathologists. Comparison between the two experts (**d**) showed very high agreement rate of 99.5%

Comparison of the grading scores of the five pathologists with the expert genitourinary pathologists score showed agreement rates between 88.5% and 97.5%. The agreement rate between the two expert pathologists was 99.5% (Fig. 2).

**Fig. 3:**
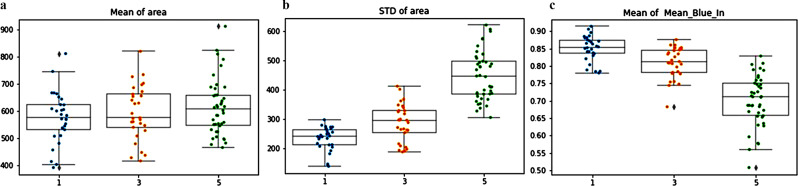
The computational analysis based nuclear features compared to the grading score of the pathologists. Tumors with grade score 1 (lowest grade) had higher mean nuclear area compared to tumors with grade score 3 and 5 (**a**). There was also a correlation between higher grade score and higher levels of standard deviation of nuclear area, a parameter that represents the nuclear pleomorphism (**b**). The parameter of the intensity of the color in the nuclei is seen (**c**), where lower levels on the scale indicate darker color. There was a correlation between higher grade score and darker nuclear color, a parameter that represents hyperchromasia

A difference of less than 2 points in the score was defined as an agreement. The algorithm based nuclear parameters were compared to the grading scores of the pathologists (Fig. 3).

**Fig. 4:**
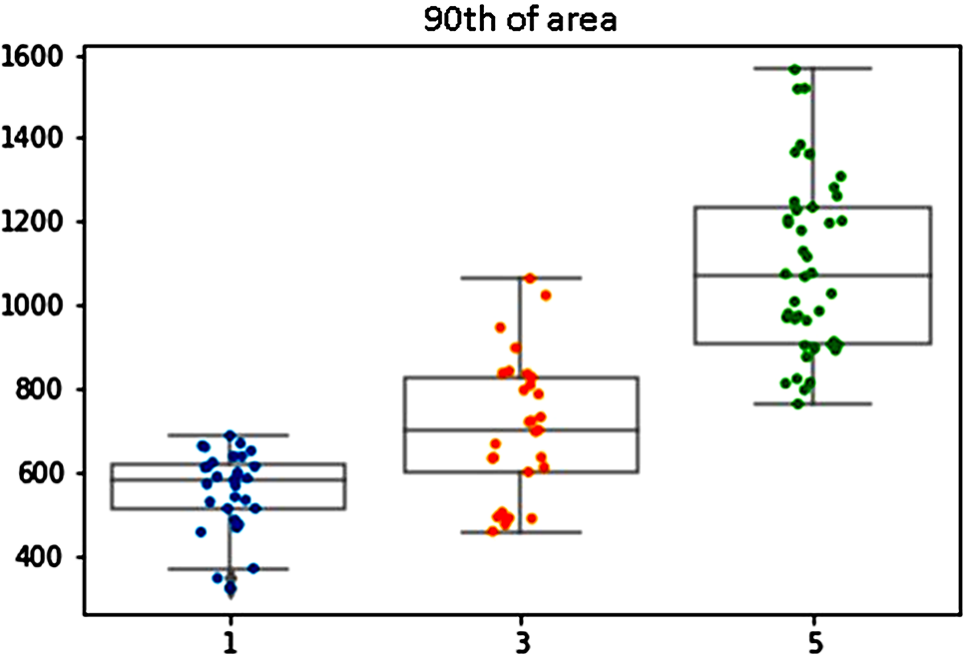
The 90th percentile of nuclear area. There was a correlation between higher tumor grade score and higher 90th percentile of nuclear area

The intuitive parameters that reflect the grade determining nuclear features indeed showed a good correlation with the grading scores of the pathologists, with agreement rates of > 85% .Tumors with grade score 5 had higher mean nuclear area (625 $\mu \text{m}$^2^) compared to tumors grade score 3 (580 $\mu \text{m}$^2^, *p* = 0.04) and grade score 1 (570 $\mu \text{m}$^2^, *p* = 0.01). This parameter represents the nuclear size. When we looked at the standard deviation of the nuclear area, there was a correlation between higher grade scores and higher levels of standard deviation of nuclear area, and this parameter reflects the nuclear pleomorphism. The intensity of the color in the nuclei also showed a correlation with the grade, with high grade tumors being associated with darker color intensity. This parameter is the measured nuclear hyperchromasia. We have also examined the 90th percentile of nuclear area (Fig. 4). The mean of the 90th percentile of nuclear area was 1050 $\mu \text{m}$^2^ for tumors in grade group 5, which as expected was significantly larger compared to tumors in grade group 3 (700 $\mu \text{m}$^2^, *p* < 0.01) and tumors in grade group 1 (592 $\mu \text{m}$^2^, *p* < 0.01).

**Fig. 5:**
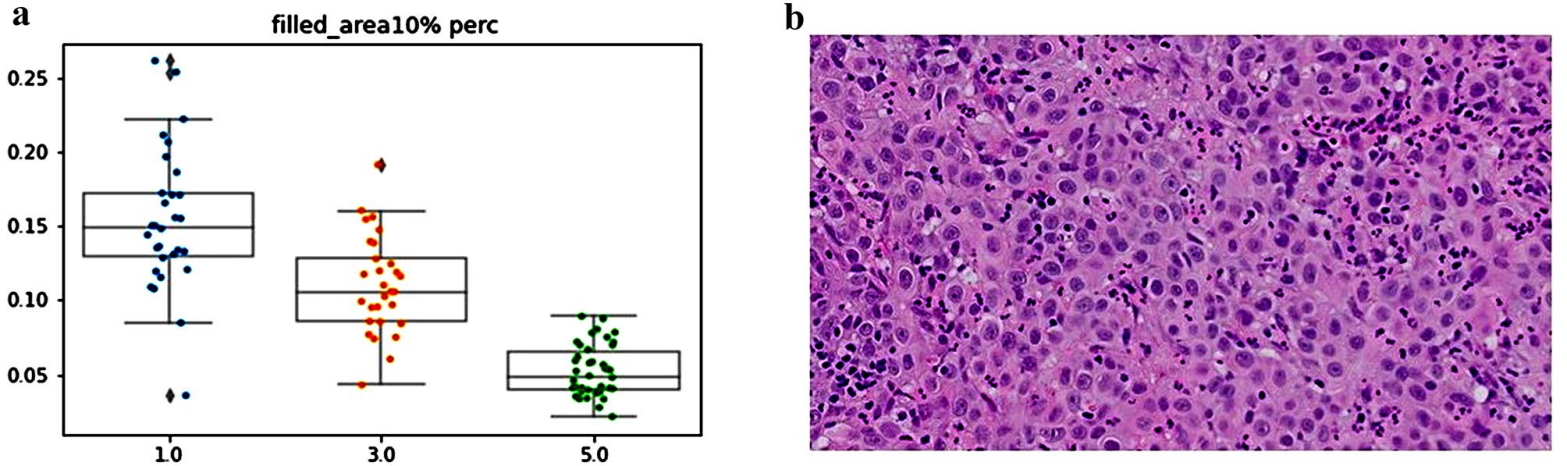
The parameter of the 10th percentile of nuclear area compared to the grading scores of the pathologists. There was a correlation between higher grade score and lower 10th percentile of nuclear area (**a**), a finding that was explained by the presence of intense inflammatory cells infiltrating between tumor cells (**b**)

Surprisingly, the parameter most associated with grade was the 10th percentile of the nuclear area. This parameter was in fact smaller in high grade tumors, meaning that the higher the tumor grade is, the smaller is the cells area in the 10th percentile. The mean of 10th percentile nuclear area was 52$\mu \text{m}$^2^ for tumors in grade group 5, which was significantly smaller compared to tumors in grade group 3 (110$\mu \text{m}$^2^, *p* < 0.01) and tumors in grade group 1 (152$\mu \text{m}$^2^, *p* < 0.01). Looking at the images we have noticed that the reason for this unexpected finding is that high grade tumors tend to have inflammatory cells (lymphocytes and neutrophils) infiltrating between tumor cells, a finding that was almost not seen at all in low grade tumors. This correlation between higher tumor grade and lower 10th percentile of nuclear area was with the highest agreement rate of 94.5% (Fig. 5).

In the training cohort, all ten cases of LGUC had almost no inflammatory cells, and only one out of the ten cases of HGUC had almost no inflammatory cells, with the other nine cases showing inflammatory infiltrate. Chi-square test showed that this difference was statistically significant (*p* < 0.01). In the validation cohort, seven out of ten (7/10) cases of HGUC had inflammation, and one out of ten (1/10) cases of LGUC had inflammation. Chi-square test also showed that this difference was statistically significant (*p* < 0.01). We have further tested the correlation between tumor grade and inflammation on a total of 100 cases by testing additional new 60 cases (30 HGUC and 30 LGUC) for the presence of inflammation. In the additional 30 HGUC cases, 19 had inflammation (19/30), and only six out of the thirty cases (6/30) of LGUC had inflammation. In the overall 100 cases, there were 35/50 HGUC with inflammation and 7/50 LGUC cases with inflammation. Chi-square test showed that the correlation between high grade and inflammation was statistically significant (*p* < 0.01).

**Fig. 6:**
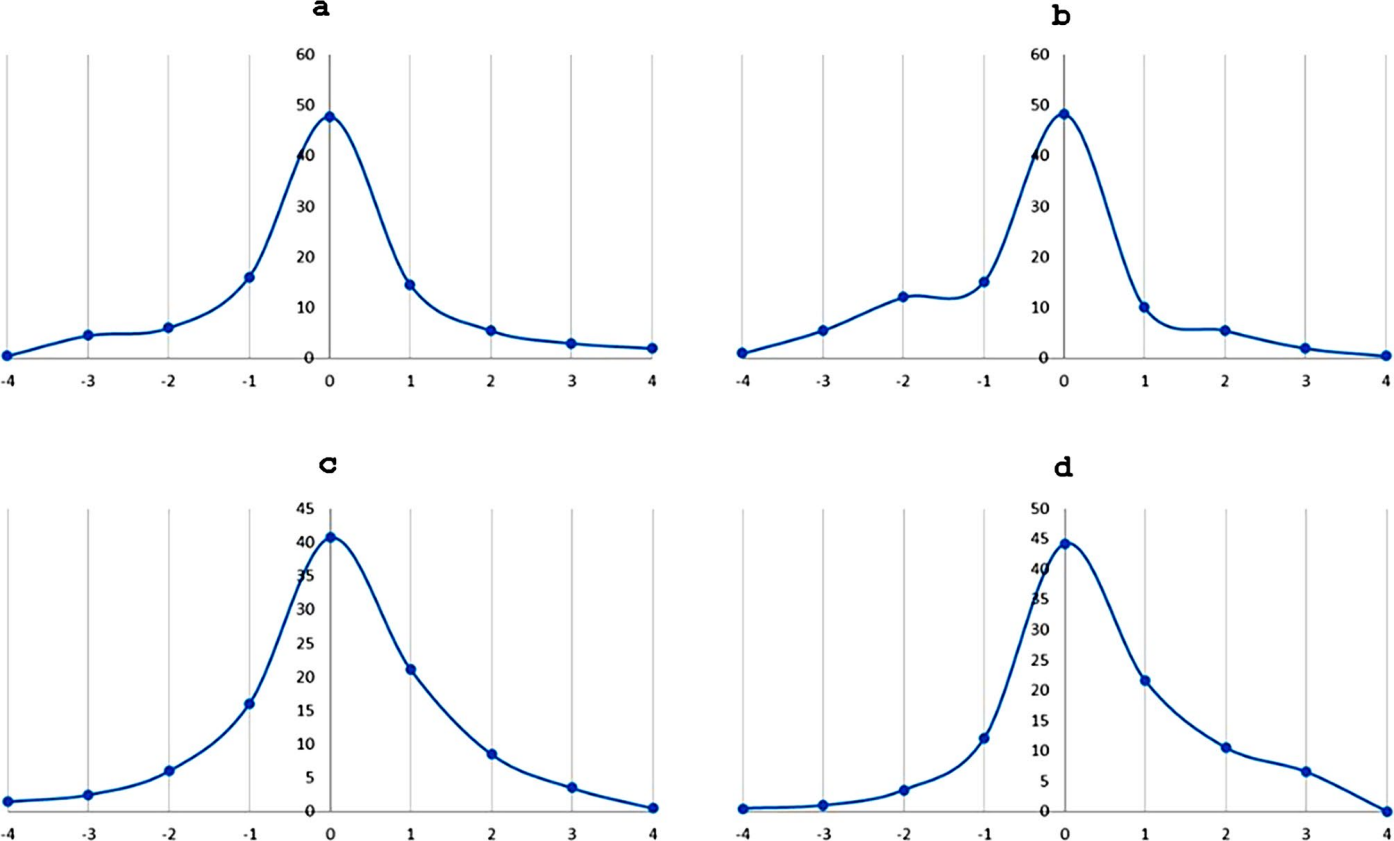
Comparison of the automated nuclear parameters of nuclear area (**a** and **c**) and color intensity (**b** and **d**) with the grading scores of the two expert genitourinary pathologists showed agreement rates of > 85%

The quantified nuclear parameters of the computer algorithm were also compared to the grading scores of the expert genitourinary pathologists, with agreement rates of > 85% (Fig. 6).

**Fig. 7:**
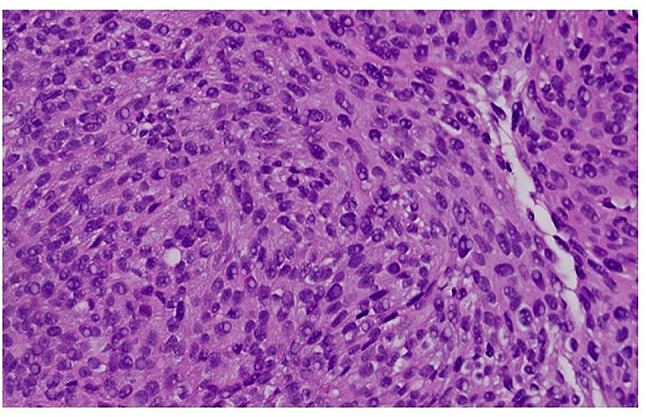
Example of a tumor with high grade nuclear features with no inflammatory cells. Such images showed disagreements between the algorithm and the expert genitourinary pathologists in the parameter of the 10th percentile of the nuclear area

Eight images showed significant difference between the algorithm and the expert pathologists. These images were from a high grade tumor with very few or no inflammatory cells (Fig. 7).

**Fig. 8:**
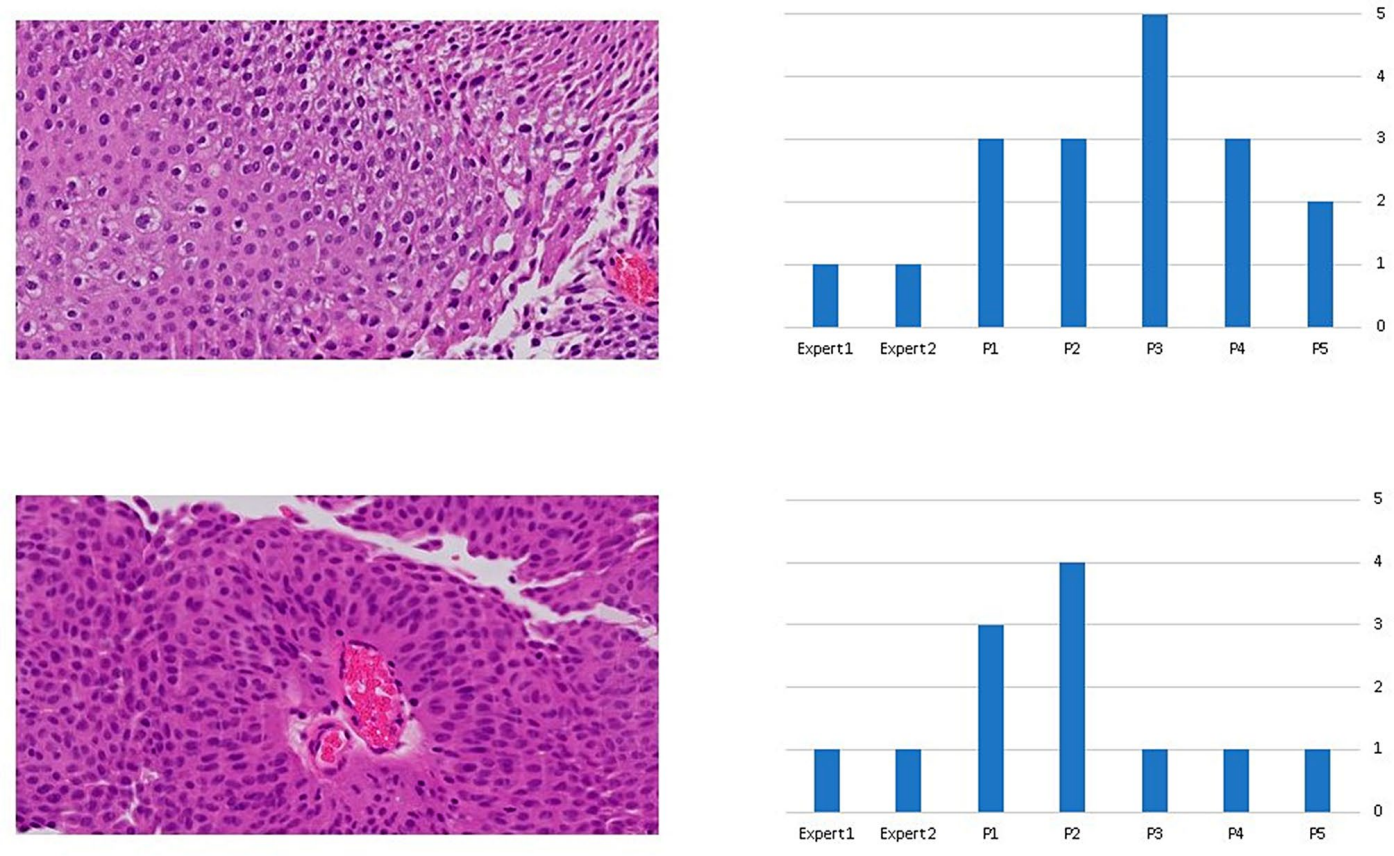
Two examples of cases showing the grading scores of the five pathologists in comparison to the grade score of the expert genitourinary pathologists. There was a tendency among the five pathologists to overgrade tumors compared to the expert pathologists

When comparing the grading scores of the five pathologists with the expert genitourinary pathologists, we have noticed that the five pathologists tended to overgrade tumors (Fig. 8).

## Discussion

Grading urothelial carcinomas is an important task for pathologists that has prognostic and therapeutic implications. Pathologists face on a daily basis grading challenges. The cytological and architectural features that are used to define the tumor grade are subjectively evaluated. Currently, there are no quantitative criteria for which a tumor passes from low grade to high grade. The grade is set according to the pathologist’s impression of the overall histological features of the tumor. Artificial intelligence and image analysis has been implemented in pathology with successful and promising results in different fields, such as Gleason score of prostate adenocarcinoma [18–21] and identification of ganglion cells to improve diagnosis of Hirschprung disease [22–24]. In this article we have analyzed the nuclear features of urothelial carcinomas. By using computational analysis tools, we transformed the grade determining nuclear features into mathematical parameters that can be measured and calculated, and then we compared these parameters with the grading scores of the pathologists. This automatic analysis showed good agreement rates when compared to five pathologists and to two expert genitourinary pathologists. We have seen that there was good correlation when examining the conventional nuclear parameters of size, pleomorphism and hyperchromasia. The added value of the computational analysis was highlighted when we looked at other parameters that are not intuitive, and what we found was that some of these parameters had the highest correlation with the grade, even better than the classic nuclear parameters that are used to define the tumor grade. The 10th percentile of the nuclear area was the parameter most associated with the grade, and as we have seen there was a correlation between higher tumor grade and lower 10th percentile of nuclear area, a finding that was explained by a histological finding of tumor infiltrating lymphocytes and neutrophils that tend to be more intense in high grade tumors compared to those with low grade. Tumor infiltrating lymphocytes have been shown to have an important role in predicting response to immunotherapy and prognosis [25, 26]. In this article, we propose a new role for inflammatory cells in urothelial carcinoma. The association between high tumor grade and intense inflammation could tip the balance toward high grade in cases where the nuclear features of the tumor cells are borderline between low and high grade. It should be noted that the parameter of the 10th percentile of nuclear area should be taken in consideration with morphology of the tumor, since it may not always represent inflammatory cells. As we have seen in this study, significant difference between the algorithm and the pathologists grading score was seen in few cases, and the reason for that was that these were tumors with high grade nuclear features and sparse inflammatory cells. A limitation of this study is that it has been performed on images of captured fields and not whole slides. When grading tumors, it is obvious that the main features pathologists look at and evaluate are those related to the tumor cells themselves, hence other cells are most often ignored. Our study highlights the importance of the tumor environment as a potential marker of tumor grade. Quantitative analysis could improve the accuracy of tumor grading and provide novel and significant insights into the biology of higher grade tumors.

## Data Availability

Te datasets used and/or analyzed during the current study are available from the corresponding author onreasonable request, pending institutional review board approval.
